# (TIMP2) x (IGFBP7) as early renal biomarker for the prediction of acute kidney injury in aortic surgery (TIGER). A single center observational study

**DOI:** 10.1371/journal.pone.0244658

**Published:** 2021-01-07

**Authors:** Jan Waskowski, Carmen A. Pfortmueller, Noelle Schenk, Roman Buehlmann, Juerg Schmidli, Gabor Erdoes, Joerg C. Schefold

**Affiliations:** 1 Department of Anesthesiology and Pain Medicine, Inselspital, Bern University Hospital, University of Bern, Bern, Switzerland; 2 Department of Intensive Care Medicine, Inselspital, Bern University Hospital, University of Bern, Bern, Switzerland; 3 Department of Cardiovascular Surgery, Inselspital, Bern University Hospital, University of Bern, Bern, Switzerland; University of Nottingham School of Medicine, UNITED KINGDOM

## Abstract

**Objective:**

Postoperative acute kidney injury (po-AKI) is frequently observed after major vascular surgery and impacts on mortality rates. Early identification of po-AKI patients using the novel urinary biomarkers insulin-like growth factor-binding-protein 7 (IGFBP7) and tissue inhibitor of metalloproteinases-2 (TIMP-2) might help in early identification of individuals at risk of AKI and enable timely introduction of preventative or therapeutic interventions with the aim of reducing the incidence of po-AKI. We investigated whether biomarker-based monitoring would allow for early detection of po-AKI in patients undergoing abdominal aortic interventions.

**Methods:**

In an investigator-initiated prospective single-center observational study in a tertiary care academic center, adult patients with emergency/ elective abdominal aortic repair were included. Patients were tested for concentrations of urinary (TIMP-2) x (IGFBP7) at baseline, after surgical interventions (PO), and in the mornings of the first postoperative day (POD1). The primary endpoint was a difference in urinary (TIMP-2) x (IGFBP7) levels at POD1 in patients with/ without po-AKI (all KDIGO stages, po-AKI until seven days after surgery). Secondary endpoints included sensitivity/ specificity analyses of previously proposed cut-off levels and clinical outcome measures (e.g. need for renal replacement therapy).

**Results:**

93 patients (n = 71 open surgery) were included. Po-AKI was observed in 33% (31/93) of patients. Urinary (TIMP-2) x (IGFBP7) levels at POD1 did not differ between patients with/ without AKI (median 0.39, interquartile range [IQR] 0.13–1.05 and median 0.23, IQR 0.14–0.53, p = .11, respectively) and PO (median 0.2, IQR 0.08–0.42, 0.18, IQR 0.09–0.46; p = .79). Higher median (TIMP-2) x (IGFBP7) levels were noted in KDIGO stage 3 pAKI patients at POD1 (3.75, IQR 1.97–6.92; p = .003). Previously proposed cutoff levels (0.3, 2) showed moderate sensitivity/ specificity (0.58/0.58 and 0.16/0.98, respectively).

**Conclusion:**

In a prospective monocentric observational study in patients after abdominal aortic repair, early assessment of urinary (TIMP-2) x (IGFBP7) did not appear to have adequate sensitivity/ specificity to identify patients that later developed postoperative AKI.

**Clinicaltrials.gov:**

NCT03469765, registered March 19, 2018.

## Introduction

Post-operative acute kidney injury (po-AKI) is often observed after cardiovascular surgery and negatively impacts on both morbidity and mortality rates [1]. AKI incidence in open abdominal aortic repair is reported to range from 20–37% [2], and increases to about 68% and 75% in suprarenal aortic aneurysm repair and ruptured aortic aneurysms, respectively [3]. In endovascular aortic repair (EVAR), AKI is noted in 9–18% of cases [3,4].

Post-operative renal (dys)-function is influenced by several factors including pre-operative renal function, co-morbidities, intraoperative factors (e.g. aortic clamping site and time, ischemia-reperfusion injury), hemodynamic instability (including vasopressor use, blood loss and need for volume replacement) and post-operative complications [5–8]. As no (or controversial) evidence for perioperative pharmacological measures to prevent po-AKI (e.g. mannitol, fenoldopam) exists [9–11], early recognition of po-AKI seems pivotal. Currently, po-AKI diagnosis is based on assessment of (course of) serum creatinine and thus estimated glomerular filtration rate (eGFR), and/or urinary output. However, respective indices may be considered insensitive in regard to timely po-AKI recognition [12].

Recently, new biomarkers including insulin-like growth factor binding protein 7 (IGFBP7) and tissue-inhibitor of metalloproteinases-2 (TIMP-2) were proposed for early detection of AKI [13]. Respective markers are considered cell cycle arrest markers and viewed to represent “renal stress”, rather than established renal damage [12]. In human kidneys, TIMP-2 is expressed in the distal nephron, whereas IGFBP7-expression is observed predominantly in the proximal tubule [14] with both markers detectable in urinary samples [12]. Both biomarkers are determined together as they showed additive value in a discovery study [13]. Moreover, both are released after a variety of possible renal insults (among others inflammation, toxins, drugs, and/or oxidative stress) [13] resulting in a potential application irrespective of underlying AKI pathology [15,16]. Data from patients with decompensated heart failure [17], major non-cardiac surgery patients [18,19], and critically ill patients [20,21] demonstrate good accuracy in predicting AKI. However, in patients undergoing cardiac surgery, data is contradictory [22–26] with a recent meta-analysis underlining the need for subsequent investigations [15]. A cut-off threshold of (TIMP-2) x (IGFBP7) <0.3 is proposed as low risk for po-AKI development, whereas patients with levels of >0.3 and <2 are regarded to have moderate AKI risk. (TIMP-2) x (IGFBP7) values >2 were previously proposed to be associated with a high risk for po-AKI development [16]. However, further research seems required before biomarker-based strategies can be implemented into clinical practice as e.g. specificity of investigated patient cohorts, potential co-medications, and other factors could affect sensitivity and specificity [27,28].

Currently, data on biomarkers for timely prediction of postoperative renal outcomes are not available for open abdominal aortic repair and EVAR patients. We therefore performed an investigator-initiated prospective observational study to investigate whether early assessment of the biomarker (TIMP-2) x (IGFBP7) would predict po-AKI in patients with abdominal aortic repair.

## Materials and methods

### Study characteristics and study patients

The *(Timp-2) x (IGfbp7) as early renal biomarker for the prediction of acute kidney injury in aortic surgery (TIGER) study* was an investigator-initiated, single center, prospective, observational study. Patients with emergency or elective abdominal aortic surgery (open repair with infra- or suprarenal clamping, or EVAR) referred to our university-based cardiovascular center were eligible for study inclusion. The study was performed from June 2018 to September 2019. Patients were excluded if any of the following criteria were met: 1) patient unable to provide informed consent (e.g. due to pre-existing severe neurological or psychiatric illness) or institutionalized patients, 2) Age < 18 years and/or 3) patients not speaking German or French. Besides additional biomarker monitoring, all patients received best standard-of-care treatment according to local standard operating procedures (SOP) without interference by the research team. The study was approved by the local Ethics Committee on Human Research (Kantonale Ethikkomission, KEK, Bern, Nr. 2018–00331). Written informed consent was obtained from all patients or respective representatives. The study was registered on Clinicaltrials.gov (NCT 03469765, registered March 19, 2018). Data quality visits (in-/exlusion criteria, informed consent, primary endpoint data) were performed by an internal qualified person.

### Study endpoints

The primary endpoint of the study was defined as a statistical significant difference in total (TIMP-2) x (IGFBP7) levels at the first postoperative day (POD1) in patients without/ with po-AKI developed in the first seven days after surgery (defined along Kidney Disease: Improving Global Outcomes (KDIGO) definitions, stages 1–3 [29]).

Secondary endpoints included A) sensitivity/specificity analyses of (TIMP-2) x (IGFBP7) along KDIGO-categories, B) comparison of the area under receiver operating characteristic curves (AUROC) for: (TIMP-2) x (IGFBP7), fractional excretion of sodium (FeNa), C) descriptive analysis of clinical endpoints including number of patients on renal replacement therapy (RRT), intensive care unit (ICU) length of stay, intermediate care unit (IMC) length of stay, hospital length of stay (LOS), and ICU-, hospital-, and 28-day-mortality. Subgroup-analyses were performed for type of surgery (open surgery, emergency and/or planned surgery). Further, the diagnostic value of absolute (Δ(TIMP-2) x (IGFBP7) 1^st^ POD = (TIMP-2) x (IGFBP7) 1^st^ POD—(TIMP-2) x (IGFBP7) baseline) and relative change (Δ (TIMP-2) x (IGFBP7) 1^st^ POD [%] = [100/(TIMP-2) x (IGFBP7) baseline] * Δ (TIMP-2) x (IGFBP7) 1^st^ POD) in urinary (TIMP-2) x (IGFBP7) was estimated.

### Biomarker assessment and patient data

Urinary and blood samples were obtained after anesthesia induction (baseline), after end of anesthesia (before IMC/ICU-admission, PO), and in the morning of the day after surgery (minimum of 8 hours up to 24 hours post-operatively, POD1). Urinary concentrations of TIMP-2 and IGFBP7 were measured using a commercially available standardized assay (NephroCheck, Astute Medical, San Diego, CA, USA). Test results are given as product of both markers in (ng/ml)^2^/ 1000. Blood and urinary creatinine and sodium concentrations were assessed to calculate fractional sodium excretion. Other post-operative laboratory parameters were assessed at the discretion of the independent treating physician.

The following pre-operative data were recorded: age; sex; BMI; American Society of Anesthesiologists (ASA) physical status classification system; comorbidities and routine medications. Laboratory parameters including serum creatinine, urea, sodium, eGFR (Chronic Kidney Disease Epidemiology Collaboration [CKD-EPI] formula) [30]. Diagnostic procedures using contrast agents (if applicable) were recorded in the 24 hours proceeding operations. The following perioperative data were recorded: type of surgery (infra- vs. suprarenal clamping, EVAR and emergency vs. elective); duration of clamping (if applicable); use/ duration of cold renal perfusion; use/ quantity of contrast agents (if applicable); duration of surgery; episodes of hypotension (mean arterial blood pressure < 20% of baseline for more than 5 minutes); cumulative dose/ type of fluids applied including autologous and allogenic packed red blood cells (RBC), fresh frozen plasma (FFP), and platelet transfusions; blood loss; use/ dose of mannitol; use/ cumulative dose of vasopressors or inotropes, and urine output during surgery. Data recorded post-operatively included routine laboratory parameters until hospital discharge (if available: sodium, potassium, serum creatinine, urea, eGFR, hemoglobin); cumulative urinary output while on IMC/ ICU; clinically overt complications after surgery (sepsis, colon ischemia, wound infection, pneumonia, delirium, others); need for and duration of RRT; length-of stay in IMC and/or ICU, length of hospital stay. ICU-/IMC-, in-hospital- and 28-day- mortality was recorded.

### Sample size calculation

An incidence of 20% po-AKI was expected in the cohort under investigation. Based on (TIMP-2) x (IGFBP7) data of previous studies in cardiac surgery patients [22,26], we assumed to reach a mean (TIMP-2) x (IGFBP7) in patients without AKI of 0.4 (ng/ml)^2^/ 1000 and in patients with AKI (i.e. KDIGO stages 1–3) a mean of 1.6 (ng/ml)^2^/ 1000 with a standard deviation (SD) of 1.4. If the true difference in the experimental and control means is 1.2, n = 18 experimental subjects and 72 control subjects are required to reject the null hypothesis that the population means of the experimental and control groups are equal with a probability (power) of 0.9. The Type I error probability associated with this test of this null hypothesis is 0.05. Assuming a drop-out rate greater than 10%, a total of 100 patients are intended to be included.

### Statistical analysis

For statistical analysis, Student’s t-test for normally distributed data and the Mann–Whitney U test for non-normally distributed data was used to compare quantitative variables between groups, ANOVA for multiple groups. Chi square test or Fisher’s exact test were used for qualitative variables, as appropriate. Statistical significance was assumed for p<0.05. Sensitivity-/specificity-analysis were performed using receiver-operating characteristics (ROC) curves using the R software package cutpointr. Diagnostic value of a biomarker is defined as excellent with Area Under the Receiver Operating Characteristics curve (AUROC) >0.9, as good with a AUROC between 0.75–0.9, as poor with a AUROC between 0.50–0.75 and as without any diagnostic value with AUROC values <0.5 [31]. Optimal cut-off values were estimated using the Youden index [31]. No optimal cut-off point was calculated for curves with poor or lower diagnostic value and for exploratory analyses. All statistical analyses were performed using the softwares SPSS for Windows (version 25;SPSS Inc., Chicago, IL, USA) and R, version 3.6.1, and Rstudio version 1.2.5001.

## Results

### Patient demographics

**Fig 1:**
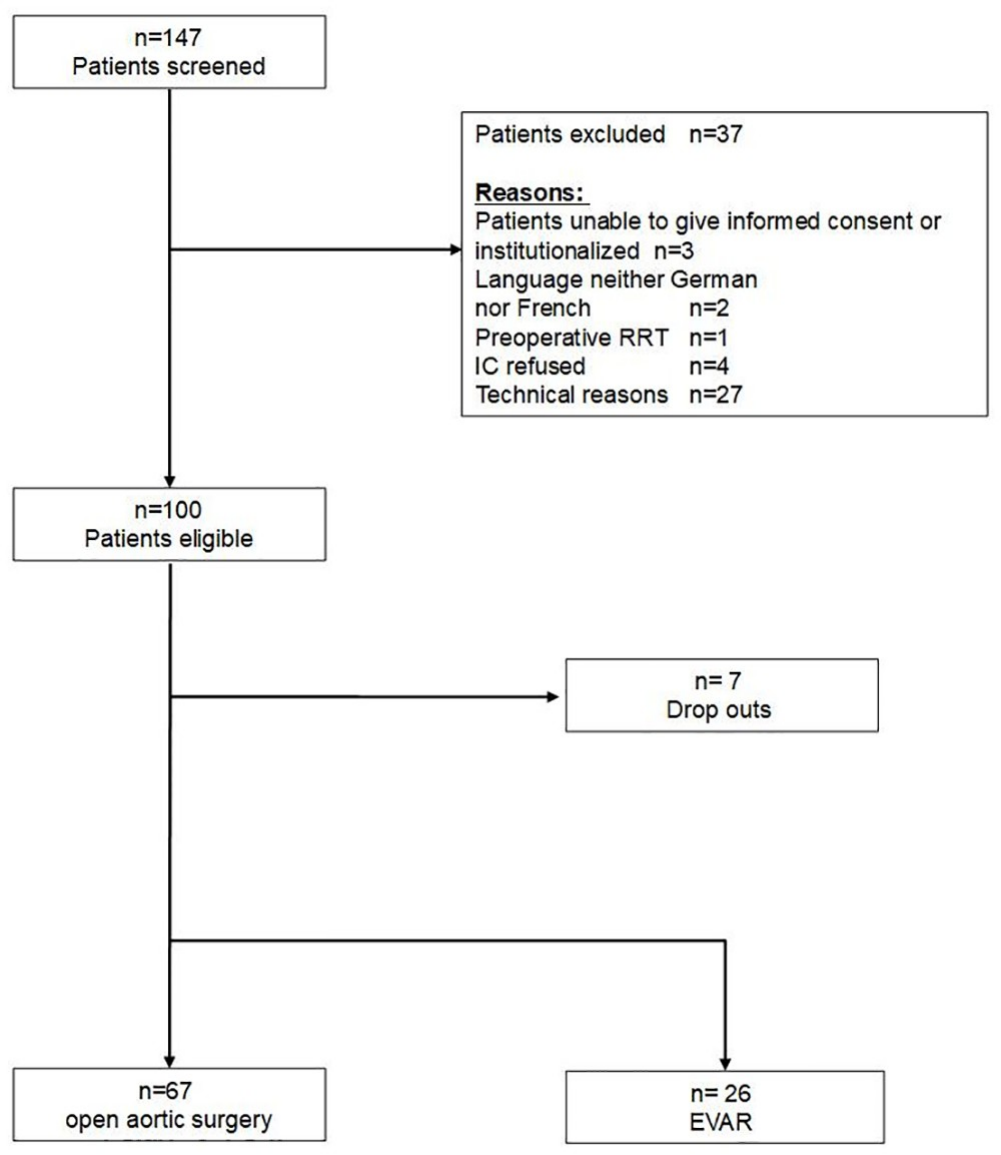
Patient flow chart. RRT Renal Replacement Therapy; IC informed consent; EVAR endovascular aortic repair.

One hundred-thirty seven patients were screened for study participation, with 100 patients eligible for study inclusion (Fig 1). Ninety-three patients were included in the final analysis set (n = 7 dropouts due to incomplete specimen sampling). As defined by KDIGO criteria, po-AKI was observed in 31 of 93 patients (33.3%) with n = 20, n = 6, n = 5 patients in KDIGO stages 1, 2, and 3, respectively. Po-AKI was diagnosed based on increased serum creatinine (n = 18), on reduced urinary output (n = 1), and based on both parameters (n = 12). Baseline demographics (Table 1) and intra-operative patient characteristics (Table 2) are given. Patients with po-AKI had higher pre-operative (104.4 ± 50.1 vs. 83.9 ± 27.4 μmol/l; p = .02) creatinine levels (Table 1). AKI patients more often had open rather than endovascular procedures, more often emergency rather than elective surgery, had longer duration of surgery, more supra- or intrarenal clamping, and received more often cold renal perfusion (all p< .05) (Table 2). Numbers of hypotensive episodes were increased in the po-AKI-group (44.3 ± 25.8 vs.27.7 ± 20.2; p = .001) despite more frequent use of vasopressors (norepinephrine) (96.8% vs. 80.6%; p = .03). Po-AKI was further associated with higher transfusion rates for autologous (87.1% vs 62.9%; p = .02) and allogeneic red blood cells (48.4% vs. 11.3%; p = .001) and fresh frozen plasma (FFPs) (45.2% vs 17.7%; p = .005). No differences were noted regarding intraoperative blood loss (p = .10) (Table 2).

**Table 1:** Baseline demographics and variables.

	Total (n = 93)	AKI group (n = 31)	No AKI group (n = 62)	p-value
**Age**	69.4 ± 9.9	71.3 ± 9.8	68.5 ± 9.9	.34
**Gender (male)**	77 (82.8%)	23(74.2%)	54 (87.1%)	.12
**BMI**	26.6 ± 4.4	27.4 ± 4.7	26.2 ± 4.3	.06
Preoperative Crea*	87 (74.25;100.75)	93 (82;122)	84 (71.5;98)	**.03**
Preoperative eGFR*	75.5 (62.25;89)	67(49;84)	81(64;90)	**.02**
Hb preoperative*	139.5 122.25;146)	130(116;146)	141(126.5;146.5)	.12
**CAD**	41 (44.1%)	13 (41.9%)	28 (45.2%)	.77
**HF >NYHA 1**	14 (15.1%)	5 (16.1%)	9 (14.5%)	.84
**Liver Cirrhosis**	2 (2.2%)	0	2(2.2%)	.31
**Diabetes**	15 (16.1%)	4 (12.9%)	11 (17.7%)	.55
**CKD**	27 (29%)	13 (41.9%)	14 (22.6%)	**.05**
**Hypertension**	71 (76.3%)	26 (83.9%)	45 (72.6%)	.23
**COPD**	22 (23.7%)	11 (35.5%)	11 (17.7%)	.06
**ASA class**				.11
**2**	4	0	4	
**3**	50	13	37	
**4**	35	16	19	
**5**	4	2	2	
**Reason for Surgery**				.51
**Aortic Aneurysm**	77 (82.8%)	26(82.9%)	51 (82.3%)	
**aorto-iliac occlusive disease**	11 (11.8%)	3 (9.7%)	8 (12.9%)	
**Graft infection**	1 (1.1%)	1(3.2%)	0	
**Other**	4 (4.3%)	1 (3.2%)	3(4.3%)	
**CM in 24h preoperative**	15(16.1%)	8 (25.8%)	7 (11.3%)	.07

Means and standard deviations (SD), medians and interquartile range (IQR) or total numbers (percentages) are given. Crea (serum) creatinine; eGFR estimated glomerular filtration rate; Hb hemoglobin; CAD coronary artery disease; COPD chronic obstructive pulmonary disease; CKD chronic kidney disease; HF heart failure; NYHA New York Heart Association Classification; ACE-I Angiotensin converting enzyme inhibitor; ARB Angiotensin II receptor blocker; BB Beta blocker; ASA American Society of Anesthesiologists physical status classification system; CM contrast media;

* n = 92 due to 1 missing value.

**Table 2:** Intraoperative patient characteristics.

	Total (n = 93)	AKI group (n = 31)	No AKI group (n = 62)	p-value
**Type of Surgery**				**.03**
Open surgery	71 (76.3%)	28 (90.3%)	43 (69.4%)	
EVAR	22 (23.7%)	3(9.7%)	19 (30.6%)	
**Re-Surgery**	10 (10.8%)	4 (12.9%)	6 (9.7%)	.64
**Urgency of surgery**				**.02**
Elective	78 (83.9%)	22 (71%)	56 (90.3%)	
Emergency	15 (16.1%)	9 (29%)	6 (9.7%)	
**Type of anesthesia**				**.03**
GA only	24 (25.8%)	13 (41.9%)	11 (17.7%)	
Combined (GA + PDA)	56 (60.2%)	17 (54.8%)	39 (62.9%)	
Regional only	4 (4.3%)	0	4 (6.5%)	
LA MAC	9 (9.7%)	1 (3.2%)	8 (12.9%)	
**Duration of surgery (min)**	293 ± 156.6	369 ± 182.2	254.9 ± 127.5	**.004**
**Type of clamping**				**.01**
None	22 (23.7%)	3 (9.7%)	19 (30.6%)	
Suprarenal	31 (33.3%)	17 (54.8%)	14 (22.6%)	
Intrarenal	2 (2.2%)	1 (3.2%)	1 (1.6%)	
Infrarenal	38 (40.9%)	10 (32.3%)	28 (45.2%)	
**Duration of total aortic clamp (min)**	81.2 ± 36.5	87.4 ± 42.3	77.2 ± 32	.23
**Cold renal perfusion**	7 (7.5%)	5 (16.1%)	2 (3.2%)	**.03**
**Number of hypotensive episodes (systolic BP ≤20% of baseline for 5 min.)**	33.3 ± 23.4	44.3 ± 25.8	27.7 ± 20.2	**.001**
Blood loss (ml)*	1950 (1550;4375)	2400 (1550;4375)	1950 (1000;2775)	.10
**Urinary output** **	520 (268;820)	590 (268;820)	520 (198;1033)	.58
**Mannitol intraoperative**	43(46.2%)	18 (58.1%)	25 (40.3%)	.11
**NA intraoperative**	80 (86%)	30 (96.8%)	50 (80.6%)	**.03**
**NA cumulative dose (μg)**	908 (515;1415)	1068 (596;1592)	745 (402;1257)	.05
**Ringer Lactate given**	5697.3±3820.2	6877.4±4249.5	5107.3±3473.9	.05
**Autologous RBC given**	66 (71%)	27 (87.1%)	39 (62.9%)	**.02**
**Vol. of autologous RBC (ml)**	964 (630;170)	1320 (744;1929)	880 (600;1290)	.08
**Allogenic RBC given**	22 (23.7%)	15 (48.4%)	7 (11.3%)	**<.0001**
**Vol. of allogenic RBC (ml)**	1200 (550;1788)	1375 (825;2200)	550 (275;1650)	.24
**FFP intraoperative**	25 (26.9%)	14 (45.2%)	11 (17.7%)	**.005**
**Vol. of FFP (ml)**	1000 (500;2250)	1125 (500;2500)	750 (500;1500)	.4

Means and SD, medians and interquartile range or total numbers (percentages) are given. EVAR endovascular aortic repair; GA general anesthesia; PDA epidural anesthesia; LA MAC local anesthesia, monitored anesthesia care; BP blood pressure; NA Noradrenalin; RBC red blood cell concentrate; FFP fresh frozen plasma; Vol. volume.

* n = 80 due to 13 missing values,

**n = 84 due to 9 missing values.

### (TIMP-2) x (IGFBP7) and AKI diagnosis

**Fig 2:**
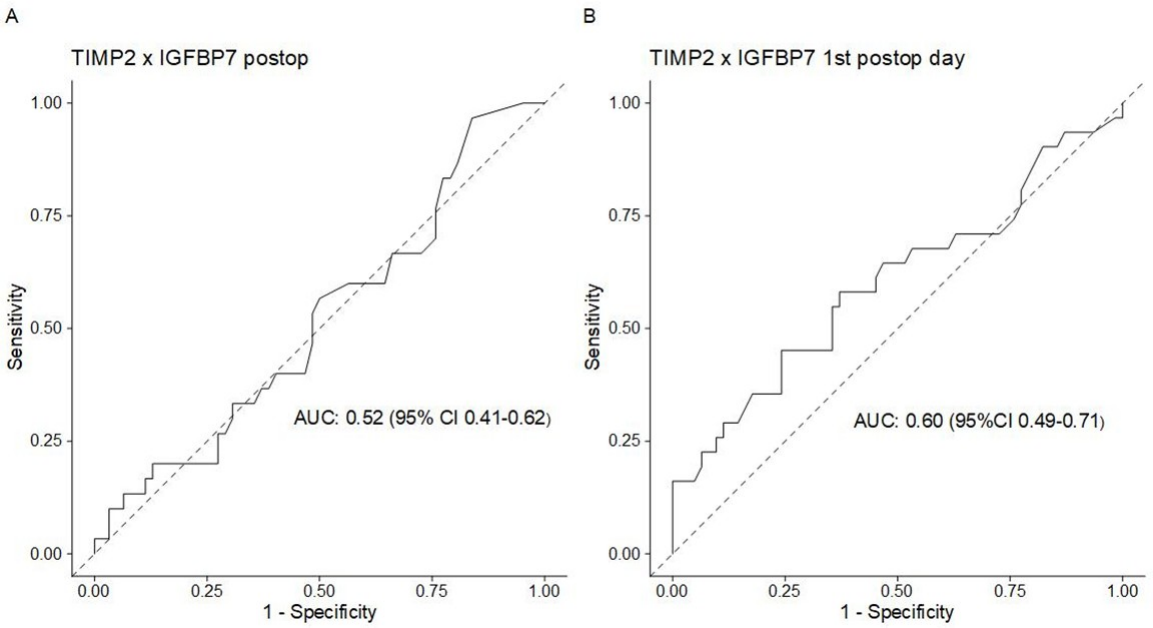
Receiver operating characteristic (ROC) curves of urinary (TIMP-2)x(IGFBP7). (A)post-operative (PO) and (B) first post-operative day (POD1) samples.

Measurement of urinary (TIMP-2) x (IGFBP7) on the morning of the first postoperative day (POD1, primary endpoint) was performed on average at 14.4 hours (± 4.3 hours) after end of surgery. No statistically significant differences were noted with regard to (TIMP-2) x (IGFBP7) levels of patients with/ without AKI on both POD1 and immediately after surgery (Table 3, S1 Fig). The AUROC-value of urinary (TIMP-2)x (IGFBP7) for POD1 assessments was 0.6 (95%-CI 0.49–0.71) and 0.52 (95%-CI 0.41–0.62) for the postoperative (PO) measurement (Fig 2). Applying the previously proposed cut-off levels of 0.3 and 2.0 to our results returned in sensitivities/specificities of 0.58/0.58 and 0.16/0.98, respectively. Negative predictive values (NPV) were 0.76 and 0.84 for testing at POD1 (for the proposed cutoffs of 0.3 and 2, respectively). For PO measurements, the previously proposed cut-offs of 0.3 and 2.0 returned in sensitivities/ specificities of 0.32/ 0.6 and 0.1/0.97, respectively (NPV 0.667 and 0.9). By using the Youden-Index we determined the following optimal cutoffs for urinary (TIMP-2) x (IGFBP7) at POD1: 0.43 (95%-CI 0.22–2.42) (sensitivity 0.45, specificity 0.65). As AUROC of urinary (TIMP-2) x (IGFBP7) was low for the immediate post-operative biomarker sample, optimal cutoffs were not tested.

**Table 3:** Primary and secondary endpoints in AKI vs. non-AKI groups.

	Total (n = 93)	AKI group (n = 31)	No AKI group (n = 62)	p-value
**Primary Endpoint**				
**(TIMP-2)x(IGFBP7) at POD1**		0.39(0.13;1.05) 0.02–8.71	0.23(0.14;0.53) 0.03–2.0	.11
**Secondary Endpoints**				
**(TIMP2)x(IGFBP7) at PO**		0.2(0.08;0.42) 0.03–6.28	0.18(0.09; 0.46) 0.02–6	.79
**FeNa PO (%)**		1.21 (0.68;2.1) 0.17–10.19	0.996 (0.58;1.83) 0.07–7.48	.30
**FeNa at POD1 (%)**		0.58 (0.26;1.16) 0.07–3.29	0.61(0.22;1.01) 0.01–4.41	.65
**LOS ICU/ IMC**	0.93 (0.79;1.86) 2.02–117.38	1.78 (0.78;4.3) 0.36–21.8	0.9 (0.79;1.8) 0.03–4.78	**.003**
**LOS Hospital**	8.96(7.02;12.02) 2.02–117.38	12.02(9.01;22.79) 3.15–117.38	7.97(3.99;9.7) 2.02–40.46)	**<.0001**
**Need for RRT**	3 (3.3%)	3 (10%)	0	
**Need for RRT on day 28**	1 (1.1%)	1 (3.2%)	0	
**Clinically overt complications**	38 (41.3%)	22 (71%)	16 (25.8%)	**<.0001**
**Sepsis**		2	0	
**Colon ischemia**		1	0	
**Wound infection**		1	3	
**Pneumonia**		1	1	
**Delirium**		2	1	
**Others and/or multiple**		17	11	
**ICU Mortality**	1 (1.1%)	1(3.2%)	0	.16
**Hospital Mortality**	1 (1.1%)	1(3.2%)	0	.16

Means and SDs and/or medians (interquartile ranges) and ranges are given, as appropriate. POD1 first post-operative day; PO postoperative; Crea (serum) creatinine; postop post-operative; FeNa fractional excretion of sodium; Hb hemoglobin; ICU intensive care unit; IMC intermediate care unit; LOS Length of stay; (TIMP2) x (IGFBP7) tissue inhibitor of metalloproteinases-2 × insulin-like growth factor binding protein-7.

Additionally, when Δ (TIMP-2) x (IGFBP7) (i.e. POD1 vs. baseline) was investigated, an AUROC of 0.63 (95%CI 0.52–0.72) for absolute and 0.65 (95%CI 0.56–0.62) for relative values of urinary (TIMP-2) x (IGFBP7) was noted.

### Clinical outcomes and fractional excretion of sodium (FeNa)

In patients with po-AKI, an early post-operative increase in serum creatinine levels after surgery was observed. No differences were noted in regard to early fractional sodium excretions between patients with vs. without AKI (Table 3). AUROC for FeNa-values immediately after surgery and on POD1 were 0.57 (95%CI 0.47–0.66) and 0.47 (95%CI 0.37–0.57), respectively.

One (3.2%) patient died in the AKI group (Table 3). Three (10%) patients required RRT postoperatively, with one (3.2%) patient in need for RRT at day 28. Longer ICU-, IMC-, and hospital stay was noted in patients with AKI (all p < .05) (Table 3). Increased rates of clinically overt postsurgical complications were noted in the AKI group (71% vs 26% without AKI; p< .0001) (Table 3).

### (TIMP-2) x (IGFBP7) levels along KDIGO AKI stages

Along KDIGO AKI stages, we observed a statistically significant difference with an increased (TIMP-2) x (IGFBP7) levels in KDIGO AKI stage 3 at POD1 only (p = .003) (Table 4). AUROC showed good diagnostic value for diagnosis of KDIGO AKI stage 3 (AUC 0.98, 95%CI 0.97–1) at POD1, but estimation based on a limited number of samples (n = 5) (indicating high risk of bias, S2 Fig). Combination of po-AKI stages 2 and 3 (as analyzed previously [13,19]) did not change the discriminatory power of the analysis (no statistical significant differences in (TIMP-2) x (IGFBP7) values of patients with (AKI stages 2+3)/ without (AKI stages 0+1) AKI on POD1 (median 0.39 vs 0.26, p = .05) (S3 Fig).

**Table 4:** Comparison of (TIMP-2) x (IGFBP7) along KDIGO AKI stages.

	KDIGO AKI stages				
	No AKI	Stage 1	Stage 2	Stage 3	p
**N**	62	20	6	5	
**Baseline**	0.66 (0.32;1.2)	0.53 (0.2;1.11)	0.6 (0.14;1.7)	0.16 (0.14;1.06)	0.28
	0.02–4.07	0.02–1.42	0.05–4.2	0.13–1.95	
**Postoperative (PO)**	0.18 (0.09;046)	0.205 (0.09;0.42)	0.08 (0.078;0.18)	0.66 (0.24;1.83)	**0.09**
	0.02–6	0.04–6.28	0.03–0.42	0.2–2.12	
**1st postop day (POD)**	0.23 (0.14;0.53)	0.35 (0.1; 0.8)	0.27 (0.11; 0.38)	3.75 (1.97;6.92)	**0.003**
	0.03–2	0.02–2.86	0.04–0.39	1.51–8.71	

Presented are medians (interquartile ranges) and ranges.

### (TIMP-2) x (IGFBP7) levels: Open surgical procedures

Seventy-one patients (76.3% of total population) received open surgery, out of which n = 28 (39.4%) had post-operative AKI (n = 18, n = 5, and n = 5 at KDIGO stages 1, 2, and 3, respectively). (TIMP-2) x (IGFBP7) levels did not differ statistically between patients with vs. without AKI immediately after the operation and at POD1 (all p>.05). Along the KDIGO stages, we observed a significant different distribution of (TIMP-2) x (IGFBP7) values with lower levels in AKI stages 0, 1, and 2 at PO (p = .04) with higher levels in KDIGO AKI stage 3 at POD1 (p = .004). Due to limited sample size, we refrained from a formal sensitivity/specificity analysis in patients with KDIGO AKI stages 2 and 3.

### (TIMP-2) x (IGFBP7) levels: Scheduled and emergency procedures

Seventy-eight patients received scheduled procedures (22 EVAR, 56 open surgery) out of which n = 22 (31%) had post-operative AKI (n = 16, n = 5, n = 1 at KDIGO stages 1, 2, and 3, respectively). No significant differences in median (TIMP-2) x (IGFBP7) levels were observed for patients with vs. without AKI (KDIGO classification) at PO and POD1 (all p>.05). Fifteen patients underwent emergency aortic surgery with po-AKI diagnosed in 9 (60%) of cases (n = 4, n = 1, and n = 4 at KDIGO stages 1, 2, and 3, respectively). In this subgroup with a limited sample size, (TIMP-2) x (IGFBP7) levels differed between patients with vs. without AKI at POD1 (p = .02) without differences at PO (p = .28). Due to limited sample size, a formal sensitivity/ specificity analysis was refrained from.

## Discussion

In an investigator-initiated prospective monocentric observational study, we did not observe differences in early postoperative (TIMP-2) x (IGFBP7) levels in abdominal aortic surgery patients with versus without po-AKI which developed in the first seven days after surgery. Higher median (TIMP-2) x (IGFBP7) levels were noted in cases of severe AKI (KDIGO stage 3). These findings were consistent along the investigated subgroups. Our findings are in contrast to previous studies demonstrating good accuracy in prediction of all-stage AKI [18,22] or KDIGO-AKI-stages 2 and 3 [13,18,19,24]. However, other authors showed that (TIMP-2) x (IGFBP7) may distinguish between patients with vs. without AKI, but with limited diagnostic accuracy following cardiac surgery [25,26].

We deliberately aimed to investigate (TIMP-2) x (IGFBP7) in patients undergoing abdominal aortic procedures as this patient group is considered at particular high risk for post-operative AKI [3]. Therefore, our patient cohort may include some heterogeneity with regard to the surgical intervention chosen (open vs. endovascular repair, emergency vs. scheduled procedures) and po-AKI etiology (inflammation, ischemia-reperfusion injury, [micro-]embolization, contrast media application, hemodynamic perturbations, and others). However, the recent literature suggests that the biomarker under investigation reflects “renal stress” induced following various renal injuries (inflammation, toxins, drugs, oxidative stress) [12]. Others have previously tested TIMP2xIGFBP7 in a variety of pathologies leading to AKI including e.g. cardiac surgery, major non-cardiac surgery, transcatheter aortic valve implantation (TAVI), critical illness, heart failure and platinum-based chemotherapy and have showed rather consistent results [17,18,20,22,23,32]. Thus, current recommendations suggest (TIMP-2) x (IGFBP7) testing in different patient cohorts irrespective of po-AKI-etiology [16], as performed in this study.

The po-AKI incidence observed (33% in overall cohort, 39% in patients receiving open aortic interventions) might be comparable to previous studies including cardiac and major non-cardiac surgery [22,24]. Of note, the subgroup with moderate to severe AKI according KDIGO (12% in our study) appeared rather comparable to other studies [22,26] or the rate is even higher [24,25]. Further, demographics and course of respective patients (including length of stay and number of postoperative complications) was also considered rather typical for AKI patients.

Recommendations for (TIMP-2) x (IGFBP7) assessment vary regarding the time of assessment [26]. We thus chose to measure (TIMP-2) x (IGFBP7) immediately after surgery and in the morning of the first postoperative day. This was performed deliberately for pragmatic reasons rather than artificial study conditions. On many if not most ICUs, respective markers would be tested at the timepoints investigated. However, earlier studies in cardiac and non-cardiac major surgery differed with regard to timing of (TIMP-2) x (IGFBP7) testing and chosen time points ranged from intra-operative testing [24], to 0 to 6 hours post-operatively [18,24–26], until post-operative day 3 [24]. Some authors tested urinary (TIMP-2) x (IGFBP7) immediately after surgery and observed decreasing (TIMP-2) x (IGFBP7) values (when compared to baseline) without differences in (TIMP-2) x (IGFBP7) levels between patients with vs. without AKI [24,25], which is in line with our findings.

Earlier studies in healthy subjects and in patients with stable chronic comorbidities showed a (TIMP-2) x (IGFBP7) reference intervals of 0.04 to 2.22 (ng/ml)^2^/ 1000 with a median at about 0.3 (and 75% <0.75 (ng/ml)^2^/ 1000) [33]. One might thus argue whether the observed increased baseline values in our study would reflect this range. Nevertheless, although we are unable to conclude on the underlying mechanisms, respective baseline levels may theoretically be related to pre-operative fasting. AKI stage 3 patients were mostly emergency patients and might theoretically have been pre-hydrated to a larger extent.

Further, timing of (TIMP-2) x (IGFBP7) may be crucial. Measurements 4–6 hours after surgery could be regarded the earliest time point for assessment [22,24]. In addition, underlying intraoperative mechanisms for AKI may differ in cardiac and vascular surgery patients (e.g. altered renal blood flow, inflammation, and haemolysis) [11], which could theoretically explain some of the differences between previous cardiac surgery studies and the current presented data. Nevertheless, in our study, the typical post-operative course of plasma creatinine and/ or decline in urinary output at 12 and 24 hours post-operatively indicated most patients with moderate to severe AKI within 14 hours. Further, 2 out of 3 patients received RRT within 24 hours. A theoretical “ideal” renal biomarker would likely diagnose moderate to severe AKI early after the impact, with good discriminatory power superior or additive to established renal biomarkers. In accordance with other studies [25,26], our data might thus challenge the early use of urinary (TIMP-2) x (IGFBP7) in patients with abdominal aortic procedures. Previously, patients with negative urinary (TIMP-2) x (IGFBP7) (<0,3[ng/ml]^2^/1000) were proposed as potential “fast track” patients [16]. Here, 50% of patients with KDIGO AKI stage 2 had levels <0.3. Thus, respective recommendations should likely be limited to cardiac surgery patients in which more data is available. However, as course of creatinine and/or urinary output indicated development of po-AKI in nearly all patients with in the first 14hours post-surgery, we theoretically might have missed maximum TIMP2xIFGBP7 concentrations. Therefore, further studies on biomarker kinetics such as the study of Cummings et al. [34] seem warranted.

Our study has important further limitations that deserve discussion. First, even if adequately powered (assumed vs. observed AKI incidence: 20% vs. 33%; assumed vs. observed drop-out rate 10% vs. 7%) to detect a difference in patients with AKI according to all KDIGO stages, the sample size of our study is limited and our results warrant larger confirmatory investigations. Second, we present data from a monocentric study with all inherent limitations. Third, pre-operative risk prediction and selection of patients (at higher risk) might have (theoretically) influenced biomarker performance [22,35]. Fourth, we assessed (TIMP-2) x (IGFBP7) at baseline and twice post-operatively. As kinetics appear not yet exactly understood, exact timing may be crucial, and more measurements might have potentially revealed a larger (temporary) rise in affected patients. Fifth, we did not test (TIMP-2) x (IGFBP7) in combination with other biomarkers e.g. urinary neutrophil gelatinase-associated lipocalin (NGAL) or peak serum cystatin C. Combining several biomarkers may improve predictive power [36]. Sixth, building of ROC curves and estimating AUROC in the subgroup of KDIGO-AKI stage 3 patients was based on a limited number of study patients. However, we aimed to account for this by bootstrapping with 1000 iterations. Seventh, in line with previous studies [22,26], this study was designed to potentially predict AKI at any AKI stage, whereas the biomarker is only validated to detect moderate to severe AKI [13].

## Conclusions

In an investigator-initiated prospective monocentric observational study, we did not observe differences in early postoperative (TIMP-2) x (IGFBP7) levels in abdominal aortic surgery patients with versus without postoperative AKI that developed in the first seven days after surgery. Subgroup analysis showed higher median (TIMP-2) x (IGFBP7) levels in patients with severe postoperative AKI (KDIGO stage 3). Further studies are required regarding the specific type of interventions and the kinetics of the biomarker.

## Supporting information

S1 Fig (TIMP2) x (IGFBP7) over time for patients with AKI/ without AKI (no AKI). Medians and IQRs are given.
(TIF)

S2 Fig ROC curves of urinary (TIMP-2)x(IGFBP7) according to KDIGO-AKI stages at the first postoperative day (POD1). (A) stage 1 vs. all other stages, (B) stage 2 vs. all other stages, (C) stage 3 vs. all other stages.
(TIF)

S3 Fig Boxplots of the subgroups of AKI stages 0+1 versus AKI stages 2+3. n.s. p .05. ^1^n = 10 for postop due to 1 missing value.
(TIF)

S1 Checklist (PDF)

S1 File (PDF)

## References

[1] Lassnigg A, Schmidlin D, Mouhieddine M, Bachmann L, Druml W, Bauer P, et al Minimal changes of serum creatinine predict prognosis in patients after cardiothoracic surgery: a prospective cohort study. J Am Soc Nephrol. 2004;15(6):1597–605. 10.1097/01.asn.0000130340.93930.dd 15153571

[2] Dariane C, Coscas R, Boulitrop C, Javerliat I, Vilaine E, Goeau-Brissonniere O, et al Acute Kidney Injury after Open Repair of Intact Abdominal Aortic Aneurysms. Annals of vascular surgery. 2017;39:294–300. 10.1016/j.avsg.2016.09.010 27890835

[3] Hobson C, Lysak N, Huber M, Scali S, Bihorac A. Epidemiology, outcomes, and management of acute kidney injury in the vascular surgery patient. Journal of Vascular Surgery. 2018;68(3):916–28. 10.1016/j.jvs.2018.05.017 30146038 PMC6236681

[4] Shahverdyan R, Majd MP, Thul R, Braun N, Gawenda M, Brunkwall J. F-EVAR does not Impair Renal Function more than Open Surgery for Juxtarenal Aortic Aneurysms: Single Centre Results. European journal of vascular and endovascular surgery: the official journal of the European Society for Vascular Surgery. 2015;50(4):432–41. 10.1016/j.ejvs.2015.04.028 26100450

[5] Schefold JC, Filippatos G, Hasenfuss G, Anker SD, von Haehling S. Heart failure and kidney dysfunction: epidemiology, mechanisms and management. Nature reviews Nephrology. 2016;12(10):610–23. 10.1038/nrneph.2016.113 27573728

[6] Godet G, Fleron MH, Vicaut E, Zubicki A, Bertrand M, Riou B, et al Risk factors for acute postoperative renal failure in thoracic or thoracoabdominal aortic surgery: a prospective study. Anesth Analg. 1997;85(6):1227–32. 10.1097/00000539-199712000-00009 9390585

[7] Dubois L, Durant C, Harrington DM, Forbes TL, Derose G, Harris JR. Technical factors are strongest predictors of postoperative renal dysfunction after open transperitoneal juxtarenal abdominal aortic aneurysm repair. Journal of vascular surgery. 2013;57(3):648–54. 10.1016/j.jvs.2012.09.043 23312936

[8] Sreeram GM, Grocott HP, White WD, Newman MF, Stafford-Smith M. Transcranial Doppler emboli count predicts rise in creatinine after coronary artery bypass graft surgery. Journal of Cardiothoracic and Vascular Anesthesia. 2004;18(5):548–51. 10.1053/j.jvca.2004.07.010 15578463

[9] Waskowski J, Pfortmueller CA, Erdoes G, Buehlmann R, Messmer AS, Luedi MM, et al. Mannitol for the Prevention of Peri-Operative Acute Kidney Injury: A Systematic Review. European journal of vascular and endovascular surgery: the official journal of the European Society for Vascular Surgery. 2019. 10.1016/j.ejvs.2019.02.003 31078413

[10] Sun H, Xie Q, Peng Z. Does Fenoldopam Protect Kidney in Cardiac Surgery? A Systemic Review and Meta-Analysis With Trial Sequential Analysis. Shock. 2019;52(3):326–33. 10.1097/SHK.0000000000001313 30601331

[11] Nadim MK, Forni LG, Bihorac A, Hobson C, Koyner JL, Shaw A, et al Cardiac and Vascular Surgery-Associated Acute Kidney Injury: The 20th International Consensus Conference of the ADQI (Acute Disease Quality Initiative) Group. J Am Heart Assoc. 2018;7(11). 10.1161/JAHA.118.008834 29858368 PMC6015369

[12] Kellum JA, Chawla LS. Cell-cycle arrest and acute kidney injury: the light and the dark sides. Nephrol Dial Transplant. 2016;31(1):16–22. 10.1093/ndt/gfv130 26044835 PMC4703048

[13] Kashani K, Al-Khafaji A, Ardiles T, Artigas A, Bagshaw SM, Bell M, et al Discovery and validation of cell cycle arrest biomarkers in human acute kidney injury. Critical care. 2013;17 10.1186/cc12503 23388612 PMC4057242

[14] Emlet DR, Pastor-Soler N, Marciszyn A, Wen X, Gomez H, William H. et al Insulin-like growth factor binding protein 7 and tissue inhibitor of metalloproteinase-2: Differential expression and secretion in human kidney tubule. Am J Physiol Renal Physiol. 2017;312:F284–F96. 10.1152/ajprenal.00271.2016 28003188 PMC5336590

[15] Liu C, Lu X, Mao Z, Kang H, Liu H, Pan L, et al The diagnostic accuracy of urinary [TIMP-2].[IGFBP7] for acute kidney injury in adults: A PRISMA-compliant meta-analysis. Medicine. 2017;96(27):e7484 10.1097/MD.0000000000007484 28682920 PMC5502193

[16] Guzzi LM, Bergler T, Binnall B, Engelman DT, Forni L, Germain MJ, et al Clinical use of [TIMP-2]•[IGFBP7] biomarker testing to assess risk of acute kidney injury in critical care: guidance from an expert panel. Critical Care. 2019;23(1):225 10.1186/s13054-019-2504-8 31221200 PMC6585126

[17] Schanz M, Shi J, Wasser C, Alscher MD, Kimmel M. Urinary [TIMP-2] x [IGFBP7] for risk prediction of acute kidney injury in decompensated heart failure. Clinical cardiology. 2017;40(7):485–91. 10.1002/clc.22683 28295429 PMC6490429

[18] Gocze I, Koch M, Renner P, Zeman F, Graf BM, Dahlke MH, et al Urinary biomarkers TIMP-2 and IGFBP7 early predict acute kidney injury after major surgery. PLoS One. 2015;10(3):e0120863 10.1371/journal.pone.0120863 25798585 PMC4370650

[19] Gunnerson KJ, Shaw AD, Chawla LS, Bihorac A, Al-Khafaji A, Kashani K, et al TIMP2*IGFBP7 biomarker panel accurately predicts acute kidney injury in high-risk surgical patients. J Trauma Acute Care Surg. 2016;80 10.1097/TA.0000000000000912 26816218 PMC4729326

[20] Bihorac A, Chawla LS, Shaw AD, Al-Khafaji A, Davison DL, Demuth GE, et al Validation of cell-cycle arrest biomarkers for acute kidney injury using clinical adjudication. American journal of respiratory and critical care medicine. 2014;189(8):932–9. 10.1164/rccm.201401-0077OC 24559465

[21] Titeca-Beauport D, Daubin D, Chelly J, Zerbib Y, Brault C, Diouf M, et al The urine biomarkers TIMP2 and IGFBP7 can identify patients who will experience severe acute kidney injury following a cardiac arrest: A prospective multicentre study. Resuscitation. 2019;141:104–10. 10.1016/j.resuscitation.2019.06.008 31216431

[22] Meersch M, Schmidt C, Aken H, Martens S, Rossaint J, Singbartl K, et al Urinary TIMP-2 and IGFBP7 as early biomarkers of acute kidney injury and renal recovery following cardiac surgery. PloS one. 2014;9 10.1371/journal.pone.0093460 24675717 PMC3968141

[23] Dusse F, Edayadiyil-Dudasova M, Thielmann M, Wendt D, Kahlert P, Demircioglu E, et al Early prediction of acute kidney injury after transapical and transaortic aortic valve implantation with urinary G1 cell cycle arrest biomarkers. BMC Anesthesiol. 2016;16:76 10.1186/s12871-016-0244-8 27609347 PMC5016985

[24] Cummings JJ, Shaw AD, Shi J, Lopez MG, O’Neal JB, Billings FTT. Intraoperative prediction of cardiac surgery-associated acute kidney injury using urinary biomarkers of cell cycle arrest. The Journal of thoracic and cardiovascular surgery. 2019;157(4):1545–53 e5. 10.1016/j.jtcvs.2018.08.090 30389130 PMC6431272

[25] Wetz AJ, Richardt EM, Wand S, Kunze N, Schotola H, Quintel M, et al Quantification of urinary TIMP-2 and IGFBP-7: an adequate diagnostic test to predict acute kidney injury after cardiac surgery? Crit Care. 2015;19:3 10.1186/s13054-014-0717-4 25560277 PMC4310039

[26] Finge T, Bertran S, Roger C, Candela D, Pereira B, Scott C, et al Interest of Urinary [TIMP-2] x [IGFBP-7] for Predicting the Occurrence of Acute Kidney Injury After Cardiac Surgery: A Gray Zone Approach. Anesth Analg. 2017;125(3):762–9. 10.1213/ANE.0000000000002116 28537976

[27] Charlton JR, Portilla D, Okusa MD. A basic science view of acute kidney injury biomarkers. Nephrology, dialysis, transplantation: official publication of the European Dialysis and Transplant Association—European Renal Association. 2014;29(7):1301–11. 10.1093/ndt/gft510 24385545 PMC4081632

[28] Teo SH, Endre ZH. Biomarkers in acute kidney injury (AKI). Best Pract Res Clin Anaesthesiol. 2017;31(3):331–44. 10.1016/j.bpa.2017.10.003 29248140

[29] Improving Global Outcomes (KDIGO) acute kidney injury work group. KDIGO clinical practice guideline for acute kidney injury. Kidney Inter Suppl. 2012;2.

[30] Levey AS, Stevens LA, Schmid CH, Zhang YL, Castro AF 3rd, Feldman HI, et al A new equation to estimate glomerular filtration rate. Annals of internal medicine. 2009;150(9):604–12. 10.7326/0003-4819-150-9-200905050-00006 19414839 PMC2763564

[31] Ray P, M.D., Ph.D., Manach Yannick L, M.D., Riou B, M.D., Ph.D., Houle Tim T, Ph.D. Statistical Evaluation of a Biomarker. Anesthesiology: The Journal of the American Society of Anesthesiologists. 2010;112(4):1023–40. 10.1097/ALN.0b013e3181d47604 20234303

[32] Schanz M, Hoferer A, Shi J, Alscher MD, Kimmel M. Urinary TIMP2IGFBP7 for the prediction of platinum-induced acute renal injury. International journal of nephrology and renovascular disease. 2017;10:175–81. 10.2147/IJNRD.S135271 28721084 PMC5500542

[33] Chindarkar NS, Chawla LS, Straseski JA, Jortani SA, Uettwiller-Geiger D, Orr RR, et al Reference intervals of urinary acute kidney injury (AKI) markers [IGFBP7][TIMP2] in apparently healthy subjects and chronic comorbid subjects without AKI. Clinica chimica acta; international journal of clinical chemistry. 2016;452:32–7. 26522657 10.1016/j.cca.2015.10.029

[34] Cummings JJ, Shaw AD, Shi J, Lopez MG, O’Neal JB, Billings FT. Intraoperative prediction of cardiac surgery–associated acute kidney injury using urinary biomarkers of cell cycle arrest. The Journal of thoracic and cardiovascular surgery. 2019;157(4):1545–53.e5. 10.1016/j.jtcvs.2018.08.090 30389130 PMC6431272

[35] Lee C-C, Chang C-H, Chen S-W, Fan P-C, Chang S-W, Chen Y-T, et al Preoperative risk assessment improves biomarker detection for predicting acute kidney injury after cardiac surgery. PLOS ONE. 2018;13(9):e0203447 10.1371/journal.pone.0203447 30180211 PMC6122821

[36] Neyra JA, Hu M-C, Minhajuddin A, Nelson GE, Ahsan SA, Toto RD, et al Kidney Tubular Damage and Functional Biomarkers in Acute Kidney Injury Following Cardiac Surgery. Kidney international reports. 2019;4(8):1131–42. 10.1016/j.ekir.2019.05.005 31440703 PMC6698294

