# Correction: (TIMP2) x (IGFBP7) as early renal biomarker for the prediction of acute kidney injury in aortic surgery (TIGER). A single center observational study

**DOI:** 10.1371/journal.pone.0259567

**Published:** 2021-10-29

**Authors:** Jan Waskowski, Carmen A. Pfortmueller, Noelle Schenk, Roman Buehlmann, Juerg Schmidli, Gabor Erdoes, Joerg C. Schefold

The following statement is missing from the Acknowledgments: An abstract of this article was published in Journal of Cardiothoracic and Vascular Anesthesia, Vol 34 S1, Waskowski J, Pfortmueller CA, Schenk N, Buehlmann R, Schmidli J, Erdoes G et al., Is (TIMP-2)X(IGFBP7) an early renal biomarker for the prediction of acute kidney injury in aortic surgery?—Results from a single center observational study, S4-S5, Copyright Elsevier (2020).

## References

[1] Waskowski J, Pfortmueller CA, Schenk N, Buehlmann R, Schmidli J, Erdoes G, et al. (2021) (TIMP2) x (IGFBP7) as early renal biomarker for the prediction of acute kidney injury in aortic surgery (TIGER). A single center observational study. PLoS ONE 16(1): e0244658. 10.1371/journal.pone.0244658 33411755 PMC7790407

