# Supplementary figures and images for: (TIMP2) x (IGFBP7) as early renal biomarker for the prediction of acute kidney injury in aortic surgery (TIGER). A single center observational study
Source: PLoS One. 2021 Jan 7;16(1):e0244658. doi: 10.1371/journal.pone.0244658 (PMC7790407; doi:10.1371/journal.pone.0244658)

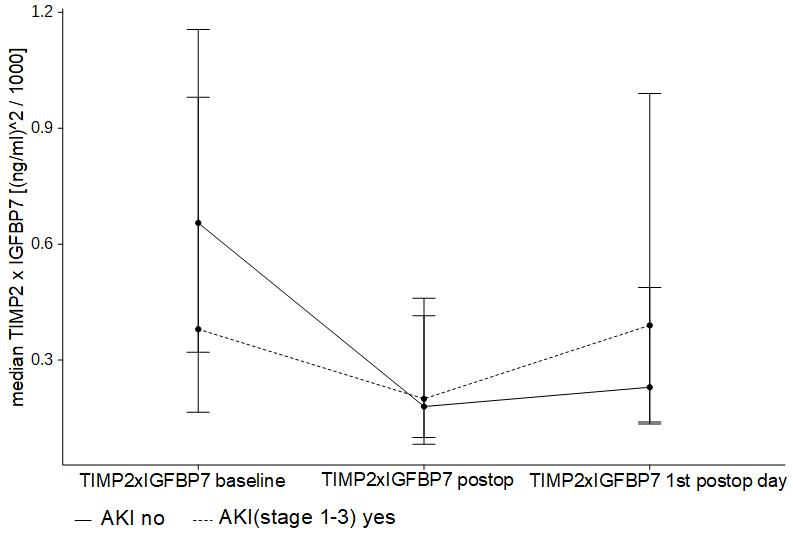

Supplement: S1 Fig — Medians and IQRs are given. (TIF) [file pone.0244658.s001.tif]

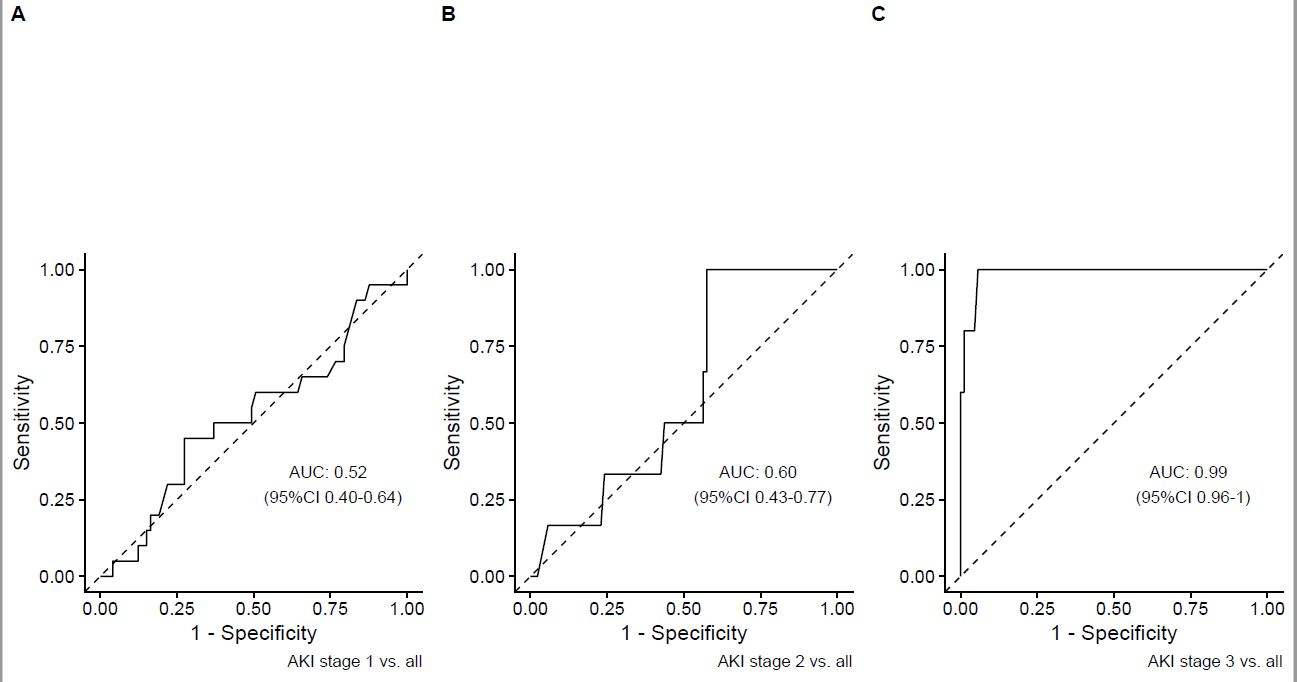

Supplement: S2 Fig — (A) stage 1 vs. all other stages, (B) stage 2 vs. all other stages, (C) stage 3 vs. all other stages. (TIF) [file pone.0244658.s002.tif]

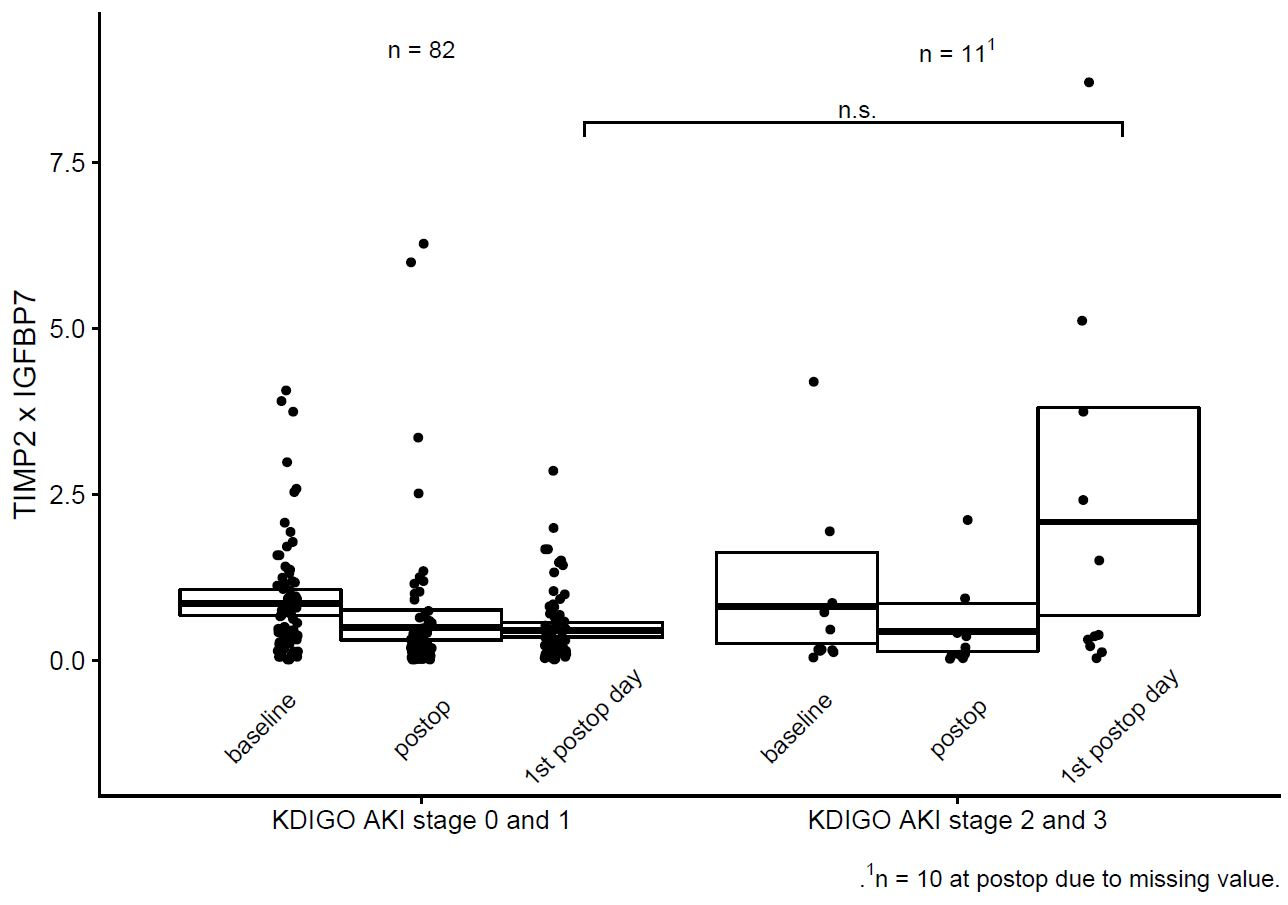

Supplement: S3 Fig — n.s. p .05. 1n = 10 for postop due to 1 missing value. (TIF) [file pone.0244658.s003.tif]
