# Supplementary material for: (TIMP2) x (IGFBP7) as early renal biomarker for the prediction of acute kidney injury in aortic surgery (TIGER). A single center observational study
Source: PLoS One. 2021 Jan 7;16(1):e0244658. doi: 10.1371/journal.pone.0244658 (PMC7790407; doi:10.1371/journal.pone.0244658)
Supplement: S1 File — (PDF) [file pone.0244658.s005.pdf]

EUROPEAN ASSOCIATION OF CARDIOTHORACIC ANAESTHESIOLOGY

35thEACTA ANNUAL CO
DECEMBER 2 - 4, 2020
GRENO
WORLD
UNITED IN DIVE

VIEW THE SUBMITTED ABSTRACT.

4. REVIEW AND CHECK OF THE SUBMITTED INFORMATION

DR. JAN WASKOWSKI (JWASKOWSKI@BLUEWIN.CH)

- In this page you can review all the data you entered through the previous steps.
- Check that all the data are correct, pay particular attention to the title and the body of the abstract: check carefull symbols, Greek letters and images you may have used.
- Check that all the abstract parts are in the correct position, before entering a symbol or an image using the table next t text area. Should this not be the case, go back to the previous page and use ctrl + X (cut) and ctrl + V (paste) in order to what you wish.
- Once everything is correct, select Continue in order to confirm the abstract submission.

EACTA2-ABS-4159-59-110870-20200416205254

Feedback from the Scientific Secretariat :

Status :	Processing ....
Received the :	16/04/2020 (dd/mm/yyyy)
Evalutation :	Not evaluated yet.

IF THE ABSTRACT WILL BE ACCEPTED, THE PRESENTATION WILL BE MADE AS FOLLOWING.

Presentation date :	
From time :	
To time :	
Where :	This data will be update after the evaluation and if the paper is accepted.
Room :	
During the session :	This data will be update after the evaluation and if the paper is accepted.

NOTES FROM THE SCIENTIFIC SECRETARIAT :

Technical notes : This data will be update after the evaluation and if the paper is accepted.
Constraint :
Other information :
Speech start time :
Speech end time :

Minutes available for presentation :
Minutes available for discussion :

LIST OF AUTHORS:

	Title	Complete first name/s	Family Name / Surname	Degree	Specialty	Job Title	Affil. n°	Pre
1.	Dr.	Jan	Waskowski	MD	Anaesthesiology	Oberarzt	1	Yes
2.	PD Dr.	Carmen Andrea	Pfortmüller	MD	Intensive Care Medicine	Oberärztin	2	
3.		Noëlle Valerie	Schenk	MSci	Intensive Care Medicine	Data Managerin	2	
4.	Dr.	Roman	Bühlmann	MD	Vascular Surgery	Spitalfacharzt	3	
5.	Prof. Dr.	Jürg	Schmidli	MD	Vascular Surgery	Chefarzt	3	
6.	Prof. Dr.	Gabor	Erdös	MD	Anaesthesiology	Leitender Arzt	1	
7.	Prof. Dr.	Joerg Christian	Schefold	MD EDIC	Intensive Care Medicine	Chefarzt	2	

LIST OF AFFILIATIONS:

	Institution	City	Coun
1.	Inselspital, Bern University Hospital, University of Bern - Department of Anesthesiology and Pain Medicine	Bern	SWIT[illegible]
2.	Inselspital, Bern University Hospital, University of Bern - Department of Intensive Care Medicine	Bern	SWIT[illegible]
3.	Inselspital, Bern University Hospital, University of Bern - Department of Cardiovascular Surgery	Bern	SWIT[illegible]

ABSTRACT CATEGORIES (TOPICS):

08 - Postoperative Care

ABSTRACT TITLE :

IS (TIMP-2)X(IGFBP7) AN EARLY RENAL BIOMARKER FOR THE PREDICTION OF ACUTE KIDNEY INJURY I[illegible] AORTIC SURGERY ? – RESULTS FROM A SINGLE CENTER OBSERVATIONAL STUDY

ABSTRACT BODY :

Introduction

Postoperative acute kidney injury (pAKI) is a typical complication after abdominal aortic repair (AAR) Urinary insulin-like growth factor-binding protein 7 (IGFBP7) and tissue inhibitor of metalloproteinas (TIMP-2) were shown to predict pAKI with good accuracy in various patient cohorts (2). In cardiac surg data is contradictory (3-5). Investigated cohorts, timing, and others may affect sensitivity/specificity (

investigated whether urinary (TIMP-2)x(IGFBP7) predicts pAKI in AAR.

Methods
Prospective, single center, observational study (NCT 03469765). Patients referred for AAR (open and endovascular interventions) were eligible. Exclusion criteria: patient unable to provide informed cons age < 18 years, not speaking German or French. We tested for concentrations of urinary (TIMP-2)x(IGF baseline, postoperative (PO) and the morning of the first postoperative day (POD). Primary outcome difference of (TIMP-2)x(IGFBP-7) in patients with/without pAKI according KDIGO (all stages) at POD.

Results
pAKI occurred in 31 of 93 (33%) patients (n=71 open surgery). (TIMP-2)x(IGFBP7) levels did not differ b patients with/ without pAKI on the first POD (median 0.39, interquartile range [IQR] 0.13-1.05 and 0.23 0.14-0.53, p= .11) and PO (0.2, IQR 0.08-0.42 and 0.18, IQR 0.09-0.46; p= .79). AUC of (TIMP-2) x (IGFBP7 0.6 (95%-CI 0.49-0.71) the first POD and 0.52 (95%-CI 0.41-0.62) PO. Higher median (TIMP-2)x(IGFBP7) occurred in pAKI KDIGO stage 3 at POD only (3.75, IQR 1.97-6.92; p= .003).

Discussion
In patients after AAR, assessment of (TIMP-2)x(IGFBP7) could not adequately predict pAKI. Further stu seem required.

References
1. Hobson C, Lysak N, Huber M, Scali S, Bihorac A. Epidemiology, outcomes, and management of acu kidney injury in the vascular surgery patient. Journal of vascular surgery. 2018;68(3):916-28.
2. Guzzi LM, Bergler T, Binnall B, Engelman DT, Forni L, Germain MJ, et al. Clinical use of [TIMP-2]•[IGF biomarker testing to assess risk of acute kidney injury in critical care: guidance from an expert panel. Care. 2019;23(1):225.
3. Meersch M, Schmidt C, Van Aken H, Martens S, Rossaint J, Singbartl K, et al. Urinary TIMP-2 and IGF early biomarkers of acute kidney injury and renal recovery following cardiac surgery. PLoS One. 2014;9(3):e93460.
4. Wetz AJ, Richardt EM, Wand S, Kunze N, Schotola H, Quintel M, et al. Quantification of urinary TIMP IGFBP-7: an adequate diagnostic test to predict acute kidney injury after cardiac surgery? Crit Care. 2015;19:3.
5. Finge T, Bertran S, Roger C, Candela D, Pereira B, Scott C, et al. Interest of Urinary [TIMP-2] x [IGFBP Predicting the Occurrence of Acute Kidney Injury After Cardiac Surgery: A Gray Zone Approach. Anest Analg. 2017;125(3):762-9.
6. Teo SH, Endre ZH. Biomarkers in acute kidney injury (AKI). Best Pract Res Clin Anaesthesiol. 2017;31(3):331-44.

Print Close

For any information, please contact: AIM Group International - Rome Office - Via Flaminia 1068 - 00189 Rome, Ital
Ph. +39-06-33053.1 - Fax +39-06-33053229 - E-mail: eactaac2020.abs@aimgroup.eu
